# Managing diabetic foot infections: a survey of Australasian infectious diseases clinicians

**DOI:** 10.1186/s13047-018-0256-3

**Published:** 2018-04-10

**Authors:** Robert J. Commons, Edward Raby, Eugene Athan, Hasan Bhally, Sharon Chen, Stephen Guy, Paul R. Ingram, Katy Lai, Chris Lemoh, Lyn-Li Lim, Laurens Manning, Spiros Miyakis, Mary O’Reilly, Adam Roberts, Marjoree Sehu, Adrienne Torda, Mauro Vicaretti, Peter A. Lazzarini

**Affiliations:** 1 Global and Tropical Health Division, Menzies School of Health Research and Charles Darwin University, PO Box 41096, Tiwi, Casuarina, Northern Territory Australia; 2 Department of Infectious Diseases, Fiona Stanley Hospital, 11 Robin Warren Dr, Murdoch, WA Australia; 3 Department of Infectious Diseases, Barwon Health, Deakin University, Geelong, VIC Australia; 4 Department of Infectious Diseases, North Shore Hospital, Waitemata District Health Board, Auckland, New Zealand; 5 Centre for Infectious Diseases and Microbiology Laboratory Services, ICPMR, Westmead Hospital, The University of Sydney, Westmead, NSW Australia; 6 Department of Infectious Diseases, Western Health, 160 Gordon St, Footscray, VIC Australia; 7 Department of Medicine, Melbourne Medical School – Western Precint, The University of Melbourne, St Albans, VIC Australia; 8 School of Pathology and Laboratory Medicine, University of Western Australia, Crawley, WA Australia; 9 Department of Infectious Diseases, John Hunter Hospital, Lookout Rd, New Lambton Heights, NSW Australia; 10 Dandenong Hospital Monash Infectious Diseases, Dandenong Hospital, 135 David St, Dandenong, VIC Australia; 11 Department of Infectious Diseases, Eastern Health, 8 Arnold St, Box Hill, VIC Australia; 12 School of Medicine and Pharmacology, University of Western Australia, Harry Perkins Research Institute, Fiona Stanley Hospital, PO Box 404, Bull Creek, WA Australia; 13 Department of Infectious Diseases, The Wollongong Hospital, Loftus St, Wollongong, NSW Australia; 14 Department of Endocrinology, University Hospital Geelong, Bellerine St, Geelong, VIC Australia; 15 Infection Management Service, Princess Alexandra Hospital, 199 Ipswich Rd, Woolloongabba, QLD Australia; 16 University of Queensland, St Lucia, QLD Australia; 17 Department of Infectious Diseases, Prince of Wales Hospital, Barker St, Randwick, NSW Australia; 18 Department of Vascular Surgery, Westmead Hospital, Hawkesbury Rd & Darcy Rd, Westmead, NSW Australia; 19 School of Clinical Sciences, Queensland University of Technology, Victoria Park Rd, Kelvin Grove, QLD Australia

**Keywords:** Diabetes mellitus, Diabetic foot infection, Australia, New Zealand, Physician, Infectious diseases

## Abstract

**Background:**

Diabetic foot infections (DFI) present a major morbidity, mortality and economic challenge for the tertiary health sector. However, lack of high quality evidence for specific treatment regimens for patients with DFIs may result in inconsistent management. This study aimed to identify DFI caseload proportion and patterns of clinical practice of Infectious Diseases (ID) Physicians and Trainees within Australia and New Zealand.

**Methods:**

A cross-sectional online survey of Australian and New Zealand ID Physicians and Trainees was undertaken, to estimate the overall ID caseload devoted to patients with DFIs and assess clinicians’ management practices of patients with DFIs.

**Results:**

Approximately 28% (142/499) of ID Physicians and Trainees from Australia and New Zealand responded to the survey. DFI made up 19.2% of all ID consultations. Involvement in multidisciplinary teams (MDT) was common as 77.5% (93/120) of those responding indicated their patients had access to an inpatient or outpatient MDT. Significant heterogeneity of antimicrobial treatments was reported, with 82 unique treatment regimens used by 102 respondents in one scenario and 76 unique treatment regimens used by 101 respondents in the second scenario. The duration of therapy and the choice of antibiotics for microorganisms isolated from superficial swabs also varied widely.

**Conclusions:**

Patients with DFIs represent a significant proportion of an ID clinician’s caseload. This should be reflected in the ID training program. Large heterogeneity in practice between clinicians reflects a lack of evidence from well-designed clinical trials for patients with DFI and highlights the need for management guidelines informed by future trials.

**Electronic supplementary material:**

The online version of this article (10.1186/s13047-018-0256-3) contains supplementary material, which is available to authorized users.

## Background

The current regional and international epidemic of diabetes mellitus has led to increasing numbers of people with diabetic foot infections (DFI) [1–4]. The 2015 Australian National Diabetes Audit found that 3.4% of patients in diabetes centres had a current foot ulcer, a two-fold increase since 2009 [5]. Diabetic foot ulcers become infected in approximately 50% of cases [6], with amputation required in over 15% [7]. A recent meta-analysis reported an estimated 3.4% of inpatients are affected by DFI [8] with foot infections now resulting in more hospitalisations than any other diabetes-related complication in Australia [9].

The creation of multidisciplinary teams (MDTs) and best practice guidelines have improved care of patients with ulcers and DFI [10] and have resulted in fewer diabetes foot-related hospitalisations and major amputations in some Australian centres [11, 12]. Current published management guidelines on DFI [13–15], however, are limited by a relative lack of published clinical trials comparing efficacy of various antimicrobial regimens [16]. Furthermore, recommendations taking into account geographic differences in the resistance rates of organisms to antimicrobials are sparse [17, 18]. Therefore, DFIs are now considered a clinical trial priority area by the Australasian Society of Infectious Diseases [19].

DiabEtic Foot Infections Australia New Zealand (DEFIANZ) is an interdisciplinary DFI interest group formed through the Australasian Society of Infectious Diseases Clinical Research Network. This preliminary study was undertaken to understand current clinical management of DFI in Australia and New Zealand and to identify areas of heterogeneity or clinical equipoise to inform future clinical trials. We aimed to identify current patterns in the clinical management of patients with DFI by Adult Infectious Diseases (ID) Physicians and Advanced Trainees in Australia and New Zealand and to determine the relative caseload of patients with DFI in this population.

## Methods

This cross-sectional study of Australian and New Zealand ID clinicians was conducted using an online survey (Survey Monkey, Palo Alto, CA, USA). Eligible participants of the survey were all Adult ID Physicians and Trainees who were currently registered and practicing in Australia or New Zealand. In 2015, there were 389 ID Physicians recorded with the Australian Health Practitioner Regulation Agency [20] and an estimated 60 Advanced Trainees. In New Zealand it is estimated there were 41 ID Physicians and 9 Trainees [21].

The survey was developed by the members of DEFIANZ and piloted by eight clinicians across Australia and New Zealand for feedback on practicality and content validity. The final items contained in the survey were agreed by consensus by the 19 members of DEFIANZ. Additional file 1 displays the final 26-item survey covering sections on demographics, clinical experience and caseloads, involvement with MDT, clinical practice compared to the Therapeutic Guidelines: Antibiotic [22], and two standardised clinical scenarios (see also Table 1).

**Table 1:** Standardised clinical scenarios from survey

Scenario 1 *Part A:* A highly functioning 63 year old lady with a history of hypertension and poorly controlled type 2 diabetes mellitus is found to have a deep heel ulcer which has been present for five weeks. She has had no previous treatment. She is afebrile with normal heart rate and blood pressure. Examination reveals a deep 2 × 3 cm ulcer with 3 cm of surrounding cellulitis and purulent discharge consistent with infection. The ulcer does not probe to bone. Peripheral pulses are present and her foot has good capillary refill, but there is evidence of peripheral neuropathy. Her white blood cell count is normal, ESR is 55 and a plain X-ray does not show osteomyelitis. A CT angiogram two months earlier revealed good arterial blood flow to both legs. She has no allergies, is a low anaesthetic risk and has normal renal function. You decide to investigate for osteomyelitis. What technique would you use (assuming all are available)? *Part B:* The imaging reveals no evidence of osteomyelitis, but evidence of deep soft tissue infection. Surgical debridement is undertaken but residual infection remains with non-debrided deep soft tissue samples growing fully sensitive *E. coli*, fully sensitive *P. aeruginosa* and methicillin sensitive *S. aureus* (MSSA) (penicillin-resistant). She is not known to be colonised by MRSA and there is a low prevalence of MRSA at your institution. Adherence is not thought likely to be an issue. What antibiotic strategy would you choose?	
Scenario 2 A highly functioning, independent 65 year old retired man with poorly controlled type 2 diabetes mellitus but no previous complications develops an ulcer overlying his 5th metatarsal head. After six weeks without treatment he attends your hospital and is found to have osteomyelitis of his 5th metatarsal head. He is afebrile with an ESR of 75. There is evidence of peripheral neuropathy and moderate peripheral arterial disease with an ankle brachial index of 0.5. A CT angiogram reveals distal small vessel disease that cannot be corrected surgically or endovascularly. He has no allergies and has normal renal function. He is not known to be colonised with MRSA and there is a low prevalence of MRSA at your institution. He has previously been adherent to oral medication and is thought to be reliable with taking medication. The patient is concerned amputation will impact on his golf and refuses amputation. He undergoes debridement of the ulcer and bone. Moderate growth of MSSA (penicillin resistant) is cultured from non-debrided deep tissue and direct microscopy reveals Gram-positive cocci. The surgeon says that there is some residual infected bone and tissue but the bone appears healthy. What antibiotic strategy would you choose?	

The Australian Therapeutic Guidelines: Antibiotic recommend empiric treatment of i) mild to moderate foot infections in patients with diabetes and no evidence of osteomyelitis or septic arthritis with amoxicillin-clavulanate or cephalexin plus metronidazole, and ii) severe limb or life threatening infection with piperacillin-tazobactam or ticarcillin-clavulanate with addition of vancomycin based on local epidemiology [22].

The primary outcome of interest was a comparative assessment of clinical management patterns of patients with DFI through two clinical scenarios. The secondary outcomes were proportional caseload attributed to inpatients and outpatients with DFI by ID clinicians and assessment of clinician involvement in MDTs.

The link to the online survey was emailed to potential participants via professional email forums with a follow-up reminder. The survey was conducted during a 4 week period in November and December 2015. The survey was also advertised weekly through the Australasian Society of Infectious Diseases electronic newsletter during the survey period. Survey responses with less than two completed items were deemed ineligible.

Statistical analysis was undertaken using Stata, version 14 (Statacorp). Descriptive statistics were used to display all variables; using proportions for categorical variables and means (standard deviations) or medians (inter-quartile ranges) for continuous variables with and without normal distributions, respectively. Calculation of summary statistics for antibiotic duration assumed the maximum duration within each response category. Respondents that answered ‘unsure’ were excluded from that question unless otherwise specified. As not all survey participants answered every question, the number of respondents answering a question was used as the denominator for the relevant results of that question. The Wilcoxon rank sum test was used to compare non-parametric continuous variables. Treatment regimens were considered unique if there was a difference in antibiotic duration or delivery method (e.g. inpatient intravenous, outpatient intravenous or oral).

## Results

A total of 159 survey responses were received. Of those, 17 were deemed ineligible and excluded, leaving a response rate of 28% (142/499) and a full completion rate of 73% (103/142). Participant characteristics are detailed in Table 2. Participants were from all Australian jurisdictions and six of 16 New Zealand regions.

**Table 2:** Survey participant characteristics and diabetic foot infection-related caseload

	Physicians		Trainees	
	No. Responses (% unless otherwise stated)	No. of respondents who answered the question	No. Responses (% unless otherwise stated)	No. of respondents who answered the question
Total number	103 (70%)	103	39 (30%)	39
Location				
Australia	77 (87%)	89	29 (94%)	31
Metropolitan (capital)	62 (81%)	77	21 (72%)	29
Urban (> 100,000)	13 (17%)	77	8 (28%)	29
Rural (< 100,000)	2 (3%)	77		
New Zealand	14 (13%)	89	2 (6%)	31
Metropolitan	4 (29%)	14	1 (50%)	2
Urban	9 (64%)	14	1 (50%)	2
Rural	1 (7%)	14		
Years of experience (median, IQR)	7 [3, 15]	103		
Year of training (median, IQR)			2 [1,3]	39
No of ID consultations per week				
Inpatient (median, IQR)	11 [6, 16]	89	21 [16.5, 30]	31
Outpatient (median, IQR)	9 [6, 16]	89	9 [6, 11]	31
No of DFI consultations per week				
Inpatient (median, IQR)	3 [2, 4]	89	5 [4, 6]	31
Outpatient (median, IQR)	2 [1, 4]	89	2 [2, 3]	31

### Caseload

Patients with DFI were estimated to represent 19.2% (586/3053) of all patients seen by responding ID clinicians per week; with patients with DFI accounting for 21.0% (197/936) of consultations by Trainees and 18.4% (389/2117) of consultations by consultant physicians. Overall inpatient caseload was 18.3% (345/1885) and outpatient caseload was 20.6% (241/1168) per week.

The most common setting for DFI consultations was as inpatients in the public hospital system (97.5%; 117/120), with 83.3% (100/120) of participants seeing DFI consultations as outpatients in the public hospital system and 19.2% of participants seeing DFI consultations in the private system. Four participants (3.3%; 4/120) could access telehealth services to also see patients. The majority of participants had on-site podiatry (93.9%; 107/114), diabetes review services (99.2%; 118/119) and vascular surgery services (88.3%; 106/120). Non-removable offloading devices (90%; 81/90) and outpatient parenteral antimicrobial therapy (99.2%; 120/121) were also commonly available.

### Multidisciplinary diabetic foot teams

Public hospital MDT were reported as available for 77.5% (93/120) of participants overall, with MDT for inpatients reported available by 50.0% (59/118) of participants and for outpatients by 59.3% (70/118) of participants. MDT consultation was reported as usually weekly or more frequently for inpatients and outpatients (65.5%; 38/58 and 65.6%; 42/64, respectively). Where MDT were available, participants often reported direct involvement (78.4%; 58/74) and attended most MDT sessions (72.7%; 40/55).

### Clinical management

Most (76.7%; 79/103) participants indicated they would treat mild to moderate DFI in accordance with Therapeutic Guidelines [22] > 60% of the time. The majority (61.2%; 63/103) of participants would prescribe antibiotics to treat all organisms isolated from superficial swabs on 40 to 80% of occasions (sometimes or often). Most (89.3%; 92/103) participants believe the MRSA prevalence rate to be 20% or less at their institution, and would therefore rarely empirically prescribe antibiotics to treat methicillin resistant *Staphylococcus aureus* (MRSA).

**Fig. 1:**
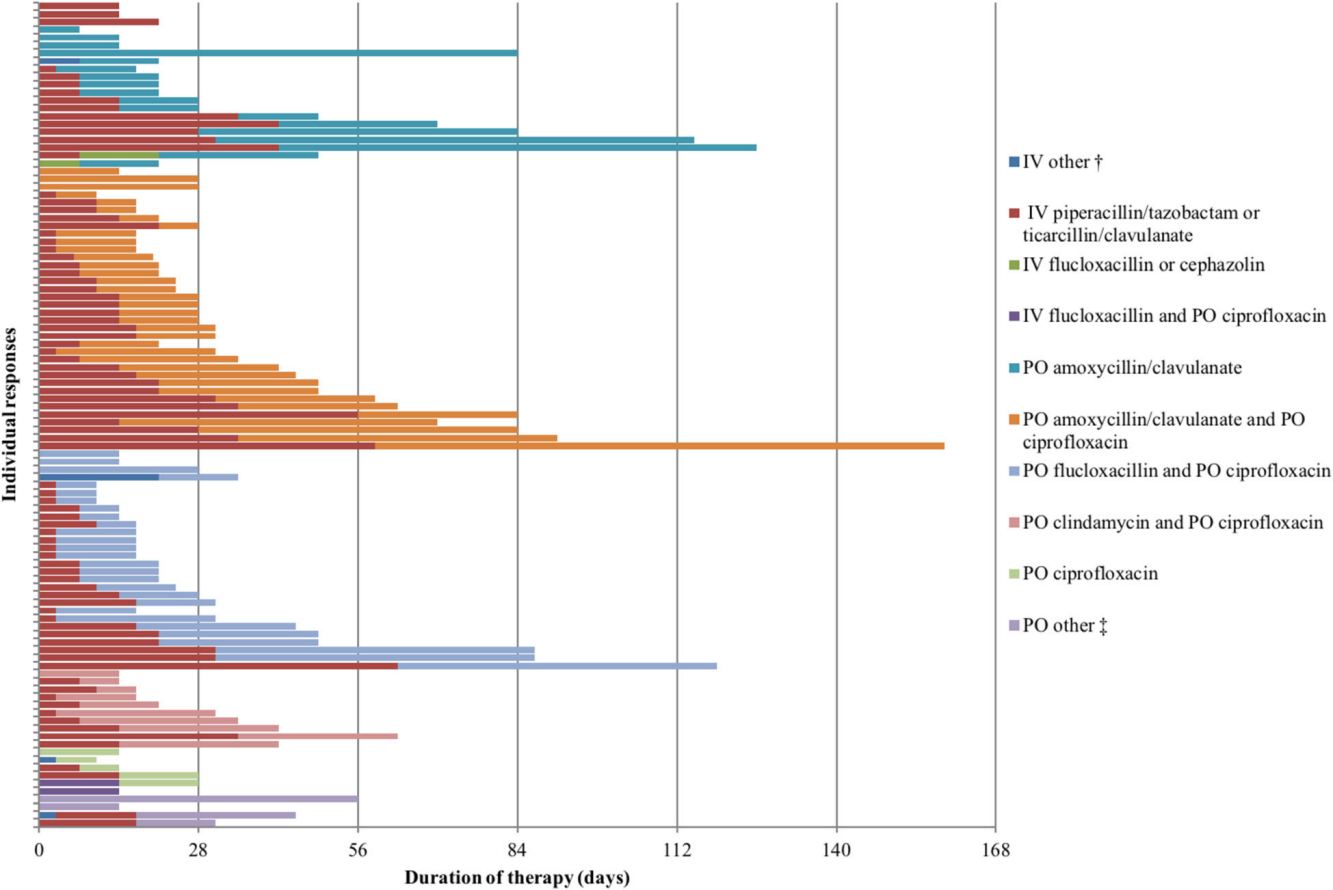
Type of antibiotics and duration chosen by respondents (*n* = 106) for Scenario 1. IV = intravenous; = PO per oral. † includes one each of ceftazidime and flucloxacillin; ceftazidime; flucloxacillin and gentamicin. ‡ includes one each of clindamycin; flucloxacillin and trimethoprim-sulfamethoxazole; trimethoprim-sulfamethoxazole and moxifloxacin; flucloxacillin

In Scenario 1 (Table 1), 82 unique antimicrobial therapy regimens were recorded by 106 participants (Fig. 1). Oral antibiotics alone were used by 13.2% (14/106) of participants, while oral antibiotics with < 4 days of intravenous antibiotics were used by a further 15.1% (16/106). Outpatient parenteral antibiotics were used by 57.5% (61/106) of participants. The median (IQR) duration of overall antibiotic therapy was 26 (17–45) days. The median duration of intravenous antibiotic therapy was 10 (3–17) days and oral antibiotic therapy without concurrent intravenous antibiotics was 14 (14–28) days. The duration of antibiotic therapy did not differ between ID physicians and Trainees (*p* = 0.93) or between clinicians in Australia and New Zealand (*p* = 0.80). Figure 1 displays the treatment regimens used for Scenario 1; 82.1% (87/106) of participants used intravenous piperacillin-tazobactam or ticarcillin-clavulanate as part of their treatment; 91.5% (97/106) covered *Pseudomonas aeruginosa* at the start of their regimen and 76.4% (81/106) of participants covered this pathogen during their entire regimen.

The preferred investigation for osteomyelitis in Scenario 1 was MRI (80%; 88/110), while CT scan was recommended by 9.1% (10/110) and bone scan by 6.4% (7/110). If methicillin sensitive *Staphylococcus aureus* (MSSA) alone was isolated from deep intraoperative specimens, 86.5% (86/104) of participants indicated they would change their treatment strategy.

**Fig. 2:**
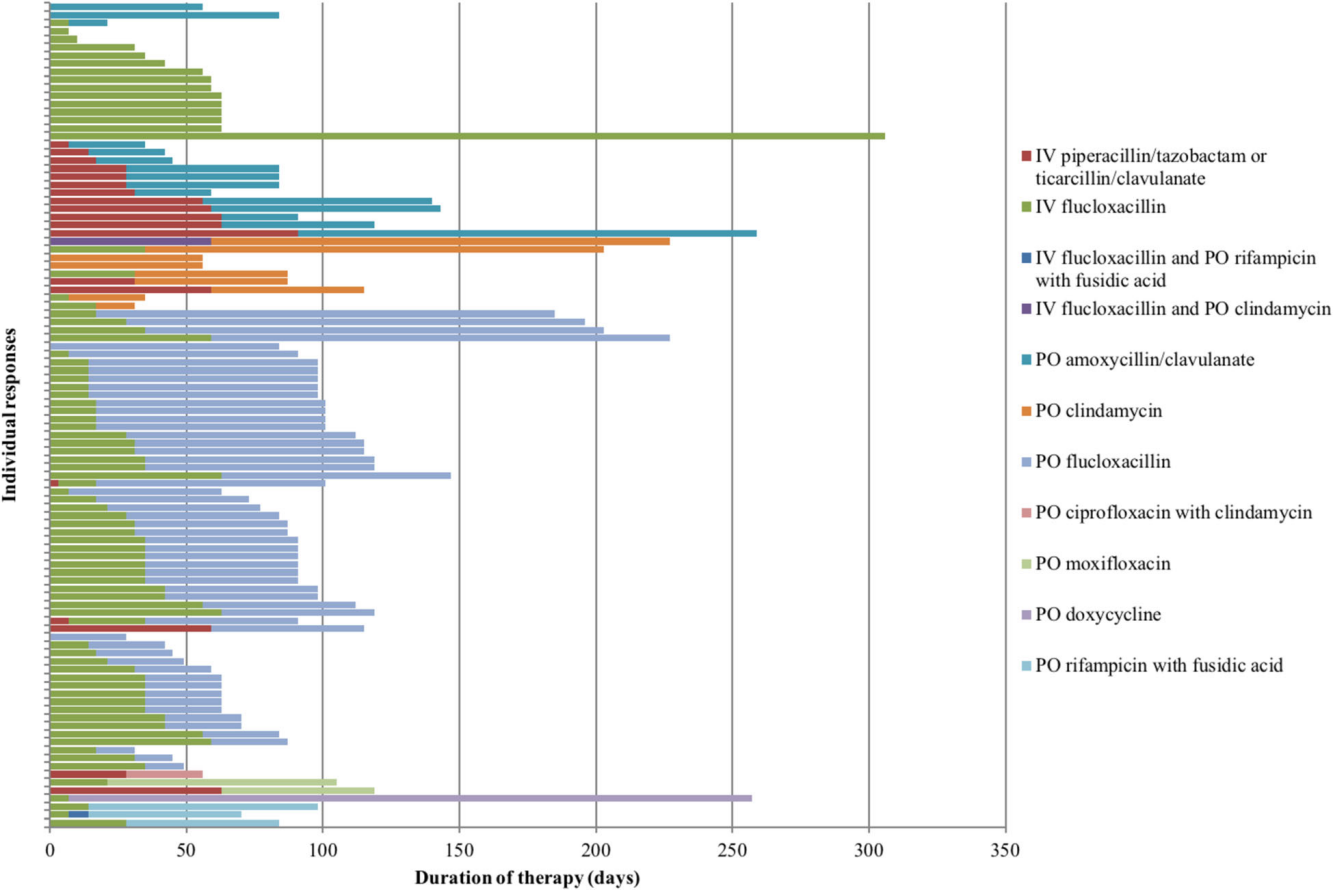
Type of antibiotics and duration chosen by respondents (*n* = 106) for Scenario 2. IV = intravenous; = PO per oral

In Scenario 2 (Table 1), 76 unique antimicrobial therapy regimens were recorded by 101 participants (Fig. 2). Oral antibiotics alone were used by 5.0% (5/101) and ≤ 14 days of intravenous antibiotics were used by 20.8% (21/101). Outpatient parenteral antibiotics were used by 85.1% (86/106) of participants. The median duration of overall antibiotic therapy was 91 (63–107) days. The median duration of intravenous therapy was 31 (17–42) days and oral therapy without concurrent intravenous antibiotics was 60 (28–90) days. There was no significant difference in duration of treatment between Physicians and Trainees (*p* = 0.73) or Australian and New Zealand clinicians (*p* = 0.21). Overall, 79.2% (80/101) gave intravenous flucloxacillin or cephazolin as part of their treatment and only 13.9% (14/101) prescribed antibiotics that would treat Gram-negative organisms during the entire duration of antibiotic treatment (Fig. 2).

## Discussion

This survey captured approximately one third of practicing ID clinicians in Australia and New Zealand and demonstrates that on average a substantial proportion (19.2%) of ID clinicians’ caseload in Australia and New Zealand relates to the management of DFI. This is in keeping with a 2012 snapshot of ID clinical practice in the region [23] and reflects the increasing burden of diabetes and its associated complications [1–4, 24]. The study also demonstrates the substantial heterogeneity that exists in ID clinicians’ approach and management of DFI. These findings highlight the need for Australian and New Zealand ID Trainees to receive increased clinical training in the management of DFI, be more involved in MDT and to assist in clinical trial design and implementation to guide development of regional evidence based guidelines that will improve the management of patients with DFI in Australasia.

Most participants managed patients with DFI as inpatients in a public hospital setting. This may reflect a higher rate of diabetes and diabetic complications in the population that attends public hospitals, increased multi-disciplinary expertise in foot-care in the public sector, the caseload of ID consultations, or simply reflect the experiences of the participants as a subgroup of total ID practice. The majority had access to outpatient parenteral antimicrobial therapy (OPAT); an effective mode of treatment that can contribute to significant healthcare savings [25]. Apart from administering intravenous antibiotics, OPAT teams are also experienced in management of complex chronic wounds and negative pressure wound therapy. While the majority of participants were familiar with OPAT to treat DFI, very few were involved in telehealth consultations. Telehealth has been demonstrated to improve diabetic foot outcomes as part of a bundle of measures, when used by clinicians at remote sites to seek advice and gain consensus opinion on difficult cases [10].

MDT management can play an important role in improving patient outcomes as part of a multi-faceted approach to DFI [10, 26–28]. Despite multiple recommendations for a uniform national model for such a service [29], a uniform model is yet to be developed. In this survey, respondents indicated MDT management was available for respondents for 50% and 59% of inpatients and outpatients, respectively. However, although the characteristics of MDTs varied significantly, the majority included ID physicians.

Local guidelines for management of DFI exist in Australasia [22]; yet the heterogeneous nature of DFI may mean that the relevant guideline may not be considered generalisable to individual patients. When directly asked about use of antibiotics for an acute infection of mild to moderate severity the majority (76.7%) of participants described using the oral antibiotics recommended in the guidelines most (> 60%) of the time. However, in response to a clinical scenario describing a patient with a five-week history of a deep ulcer (categorised as an infection of moderate severity), only 13.2% of respondents used oral therapy and a further 15.1% used a course of intravenous antibiotics less than 4 days followed by oral therapy. As such, 71.7% respondents who used a more prolonged intravenous course were not complying with the suggested Australian guidelines [22]. This result highlights the difficulties in determining appropriate management in DFI, in this case in classifying the severity of the infection, in order to discriminate treatment modalities or treatment durations using current guidelines. It is also possible that clinicians may be unaware of the guidelines, or sceptical of the value of guidelines in view of their lack of evidence base. In the other clinical scenario, the challenge in applying current guidelines was also demonstrated, with participants choosing a range of different treatment options which would be consistent with both acute and chronic osteomyelitis.

The heterogeneity of the treatment regimens prescribed by respondents likely reflects the lack of consensus in management of DFI. This is most likely due to a combination of:

i) a lack of high quality randomised controlled trial-based evidence to inform guidelines;
ii) the heterogeneous nature of DFI that not only restricts enrolment of uniform patients into clinical trials but potentially prevents clinical trial results from being generalisable;
iii) the multiple confounding factors that impact the efficacy of antibiotics in the management of DFIs, including difficulties in source control, poor supply of nutrients and antibiotics due to micro- and macrovascular factors and compliance with the medical care bundle including orthotic use.
iv) the spectrum of DFI outcomes where some patients will inevitably fail to heal. Such failure to heal risks retrospectively being blamed upon early antibiotic cessation or inadequate antimicrobial coverage, which may pre-emptively influence a clinician’s judgement as to the best antibiotic regimen and duration; and
v) a lack of awareness of the grading and management of DFI according to published guidelines.

The heterogeneity in recommended management, such as oral versus intravenous antibiotic administration, demonstrates clinical equipoise, supporting the ethical argument for performance of randomised controlled trials studying antimicrobial management of DFI.

Despite considerable heterogeneity in management there were some areas of consistency: i) MRI was considered the preferred option to investigate potential osteomyelitis; ii) the majority of clinicians in hospitals with a perceived low or intermediate prevalence of MRSA (< 20%) do not cover MRSA empirically and iii) intravenous followed by oral flucloxacillin was the treatment of choice for patients with MSSA osteomyelitis, with only 13.9% treating for Gram-negative organisms despite the ulcer having been present for 6 weeks.

Whether to treat organisms found on superficial swabs remains a difficult decision, with respondents divided as to whether they would cover organisms obtained from superficial swabs. Sampling for microbiological specimens should ideally be uniform with deep tissue samples collected and processed in a standardised manner [30]. Our experience suggests this is uncommon and superficial swabs may be the only microbiological specimens that an ID clinician has to base management decisions upon. However, as demonstrated in Clinical Scenario 1, when well-collected samples only cultured MSSA, the majority of clinicians (86.5%) would adjust antibiotics based upon this.

This study has limitations. There is a high likelihood of a response bias, with clinicians that commonly see such patients being more inclined to participate in this survey. However, this is offset by a response rate of nearly one third of all practicing ID clinicians, the proportional uniformity of the caseload across different categories of respondents and the similarities to a previous study that found 19% of ID consultations from the region were for people with diabetes [23]. As the majority of the respondents in this study worked in the public sector, our data may not be generalisable to physicians practicing in private. Lastly, scenario-based assessment of clinical management is limited by difficulties in replicating the multiple variables that impact on a clinician’s management decision, as well as the clinical response at different time points that may change an initial management plan. However, the heterogeneity in treatment regimens in this survey reflects real-world clinical experience and the complexity of this patient group. These results could be supported by additional cohort study data, that may provide further evidence of heterogeneity in clinical practice.

## Conclusions

This study found nearly one in every five consultations provided by Australian and New Zealand ID Physicians and Trainees were for patients with DFI, and that the treatment recommended is heterogeneous. The study highlights the need for outcome-directed randomised clinical trials. Patients with DFI currently form an integral part of an ID Physician’s practice and because this is likely to increase in the future, the ID community needs to continue to recognise the importance of DFI as a significant component of ID practice and training.

## Additional file

Additional file 1: Questionnaire. (DOCX 34 kb)

## Abbreviations

CI: Confidence Interval
CT: Computed tomography
DEFIANZ: DiabEtic Foot Infections Australia New Zealand
DFI: Diabetic foot infections
ID: Infectious Diseases
IQR: Interquartile Range
MDT: Multidisciplinary teams
MRI: Magnetic resonance imaging
MRSA: Methicillin resistant *Staphylococcus aureus*
OPAT: Outpatient parenteral antimicrobial therapy
