# Supplementary material for: Managing diabetic foot infections: a survey of Australasian infectious diseases clinicians
Source: J Foot Ankle Res. 2018 Apr 10;11:13. doi: 10.1186/s13047-018-0256-3 (PMC5894166; doi:10.1186/s13047-018-0256-3)
Supplement: Supplementary file 1 — Questionnaire. (DOCX 34 kb) [file 13047_2018_256_MOESM1_ESM.docx]

**Additional Files**

**Additional File 1: Questionnaire**

**Job description**

1. What is your current position?

- Infectious Diseases Trainee
- Infectious Diseases Consultant
- Other

**Advanced Infectious Diseases Trainees**

2. How many years of Advanced Infectious Diseases Training have you entirely completed?

**Infectious Diseases Physicians**

3. How many years of consultant practice as an Infectious Diseases Physician have you entirely completed?

**Baseline Data**

4. In which country and region do you work?

5. What best describes the setting of your main place of practice?

- Metropolitan Area (Capital City)
- Urban (>100,000 population, not Capital city)
- Rural (centres with population <100,000 people)

6. How many infectious diseases inpatients do you see in an average week under your own care or as consults for another team? (Please average your numbers for ward service and non-ward service weeks if applicable)

7. How many infectious diseases outpatients do you see in an average week, including patients on outpatient parenteral therapy? (Please average your numbers for ward service and non-ward service weeks if applicable)

8. How many inpatients with diabetic foot infections (e.g. cellulitis, infected ulcers or osteomyelitis) do you see in an average week under your own care or as consults for another team? (Please average your numbers for ward service and non-ward service weeks if applicable)

9. How many outpatients with diabetic foot infections (e.g. cellulitis, infected ulcers or osteomyelitis) do you see in an average week, including patients on outpatient parenteral therapy? (Please average your numbers for ward service and non-ward service weeks if applicable)

10. In a given month, in what setting do you see patients with diabetic foot infections? (Please provide an approximate percentage of total patients with diabetic foot infections for each category; the total must be 100%)

- Public hospital inpatients
- Public hospital outpatients (including outpatient parental therapy/ Hospital in the Home)
- Private hospital inpatients
- Private outpatients (including outpatient parental therapy/ Hospital in the Home)
- Community clinic
- Telehealth
- Other

11. Do patients with diabetic foot infections at your main practice have on-site access to the following?

- Multidisciplinary Team (MDT) reviews
- Vascular surgery consultation
- Podiatry review
- Diabetes review
- Non removable casts
- Outpatient parenteral antibiotic delivery

12. Does your public hospital have a multidisciplinary team for patients with diabetic foot disease?

- Yes - Inpatient only
- Yes - Outpatient only
- Yes - Inpatient and outpatient
- No
- Unsure
- I do not work in a public hospital

**Multidisciplinary Teams**

Answer all questions for diabetic foot multidisciplinary teams (MDTs) at your main public hospital of practice.

13. How does the MDT predominantly operate?

- Multiple disciplines review patients in the same clinic or on the same round
- Multiple disciplines review patients at different times but meet as a team to discuss management
- Disciplines see patients in different clinics or at different times and liaise as needed
- My public hospital does not have a multidisciplinary diabetic foot team (please skip to question 16)
- Other (please specify)

14. What disciplines are routinely involved in the MDT?

- Diabetic educators
- Endocrinology
- General Medicine
- General Surgery
- Infectious Diseases
- Microbiology / Clinical Microbiology
- Nursing
- Podiatry
- Psychologist
- Renal
- Social work
- Surgery: Orthopaedic
- Surgery: Vascular
- Other (please specify)

15. How often does the MDT operate?

16. At what percentage of MDT sessions are Infectious Diseases Physicians or Trainees present?

- 0
- >0 to 25
- >25 to 50
- >50 to 75
- >75 to <100
- 100
- Unsure

**Scenario 1**

A highly functioning 63 year old lady with a history of hypertension and poorly controlled type 2 diabetes mellitus is found to have a deep heel ulcer which has been present for five weeks. She has had no previous treatment. She is afebrile with normal heart rate and blood pressure. Examination reveals a deep 2 x 3cm ulcer with 3cm of surrounding cellulitis and purulent discharge consistent with infection. The ulcer does not probe to bone. Peripheral pulses are present and her foot has good capillary refill, but there is evidence of peripheral neuropathy.

Her white blood cell count is normal, ESR is 55 and a plain X-ray does not show osteomyelitis. A CT angiogram two months earlier revealed good arterial blood flow to both legs. She has no allergies, is a low anaesthetic risk and has normal renal function.

17. You decide to investigate for osteomyelitis. What technique would you use (assuming all are available)?

- Bone scan
- CT
- Gallium scan
- MRI
- White cell scan
- Other (please specify)

18. The imaging reveals no evidence of osteomyelitis, but evidence of deep soft tissue infection. Surgical debridement is undertaken but residual infection remains with non-debrided deep soft tissue samples growing fully sensitive E. coli, fully sensitive P. aeruginosa and methicillin sensitive S. aureus (MSSA) (penicillin-resistant). She is not known to be colonised by MRSA and there is a low prevalence of MRSA at your institution. Adherence is not thought likely to be an issue. What antibiotic strategy would you choose?

- Intravenous course only
- Initial intravenous course followed by oral therapy
- Concurrent intravenous and oral therapy or concurrent therapy followed by ongoing oral therapy
- Oral course only

***Scenario 1 - Intravenous antibiotics only***

You have chosen to give intravenous antibiotics only. Please identify below whether these would be given as an inpatient, outpatient or combination of these locations and what antibiotics you would use for what duration.

19. What intravenous antibiotic(s) would you give as an inpatient?

- None
- Amoxycillin/Ampicillin
- Amoxycillin/clavulanate
- Benzylpenicillin
- Ceftaroline
- Ceftazidime
- Ceftriaxone
- Cefuroxime
- Cephazolin
- Clindamycin
- Flucloxacillin
- Ertapenem
- Gentamicin
- Lincomycin
- Linezolid
- Metronidazole
- Meropenem
- Piperacillin/tazobactam
- Ticarcillin/clavulanate
- Vancomycin

20. What would be the likely duration of inpatient intravenous antibiotics?

- 0 days
- 1 day to 3 days
- 4 days to 7 days
- 8 days to 14 days
- 15 days to 28 days
- >1 month to 2 months
- >2 months to 3 months
- >3 months to 6 months
- >6 months

21. What intravenous antibiotic(s) would you give as an outpatient? (assumed to follow inpatient intravenous therapy unless 0 days of inpatient therapy are given)

- None
- Amoxycillin/Ampicillin
- Amoxycillin/clavulanate
- Benzylpenicillin
- Ceftaroline
- Ceftazidime
- Ceftriaxone
- Cefuroxime
- Cephazolin
- Clindamycin
- Flucloxacillin
- Ertapenem
- Gentamicin
- Lincomycin
- Linezolid
- Metronidazole
- Meropenem
- Piperacillin/tazobactam
- Ticarcillin/clavulanate
- Vancomycin

22. What would be the likely duration of outpatient intravenous antibiotics?

- 0 days
- 1 day to 3 days
- 4 days to 7 days
- 8 days to 14 days
- 15 days to 28 days
- >1 month to 2 months
- >2 months to 3 months
- >3 months to 6 months
- >6 months

***Scenario 1 - Intravenous antibiotics followed by oral antibiotics***

You have chosen to give intravenous antibiotics initially followed by oral antibiotics. Please identify below whether the intravenous antibiotics would be given as an inpatient, outpatient or combination of these locations and what antibiotics you would use for what duration.

23. What intravenous antibiotic(s) would you give as an inpatient?

- None
- Amoxycillin/Ampicillin
- Amoxycillin/clavulanate
- Benzylpenicillin
- Ceftaroline
- Ceftazidime
- Ceftriaxone
- Cefuroxime
- Cephazolin
- Clindamycin
- Flucloxacillin
- Ertapenem
- Gentamicin
- Lincomycin
- Linezolid
- Metronidazole
- Meropenem
- Piperacillin/tazobactam
- Ticarcillin/clavulanate
- Vancomycin

24. What would be the likely duration of inpatient intravenous antibiotics?

- 0 days
- 1 day to 3 days
- 4 days to 7 days
- 8 days to 14 days
- 15 days to 28 days
- >1 month to 2 months
- >2 months to 3 months
- >3 months to 6 months
- >6 months

25. What intravenous antibiotic(s) would you give as an outpatient? (assumed to follow inpatient intravenous therapy unless 0 days of inpatient therapy are given)

- None
- Amoxycillin/Ampicillin
- Amoxycillin/clavulanate
- Benzylpenicillin
- Ceftaroline
- Ceftazidime
- Ceftriaxone
- Cefuroxime
- Cephazolin
- Clindamycin
- Flucloxacillin
- Ertapenem
- Gentamicin
- Lincomycin
- Linezolid
- Metronidazole
- Meropenem
- Piperacillin/tazobactam
- Ticarcillin/clavulanate
- Vancomycin

26. What would be the likely duration of outpatient intravenous antibiotics?

- 0 days
- 1 day to 3 days
- 4 days to 7 days
- 8 days to 14 days
- 15 days to 28 days
- >1 month to 2 months
- >2 months to 3 months
- >3 months to 6 months
- >6 months

27. What oral antibiotics would you give after the intravenous antibiotics? (Check all that apply)

- Amoxicillin
- Amoxycillin/ clavulanate
- Cephalexin
- Cefuroxime
- Ciprofloxacin
- Clindamycin
- Cotrimoxazole
- Di/Flucloxacillin
- Doxycycline
- Fusidic Acid
- Linezolid
- Metronidazole
- Moxifloxacin
- Phenoxymethylpenicillin
- Pristinamycin
- Rifampicin

28. What would be the likely duration of the oral antibiotics?

- 0 days
- 1 day to 3 days
- 4 days to 7 days
- 8 days to 14 days
- 15 days to 28 days
- >1 month to 2 months
- >2 months to 3 months
- >3 months to 6 months
- >6 months

***Scenario 1 - Concurrent intravenous antibiotics and oral antibiotics***

You have chosen to give concurrent intravenous and oral antibiotics, or intravenous antibiotics followed by concurrent antibiotics. Please identify below whether the intravenous antibiotics would be given as an inpatient, outpatient or combination of these locations and what antibiotics you would use for what duration.

29. What intravenous antibiotic(s) would you give as an inpatient?

- None
- Amoxycillin/Ampicillin
- Amoxycillin/clavulanate
- Benzylpenicillin
- Ceftaroline
- Ceftazidime
- Ceftriaxone
- Cefuroxime
- Cephazolin
- Clindamycin
- Flucloxacillin
- Ertapenem
- Gentamicin
- Lincomycin
- Linezolid
- Metronidazole
- Meropenem
- Piperacillin/tazobactam
- Ticarcillin/clavulanate
- Vancomycin

30. What would be the likely duration of inpatient intravenous antibiotics?

- 0 days
- 1 day to 3 days
- 4 days to 7 days
- 8 days to 14 days
- 15 days to 28 days
- >1 month to 2 months
- >2 months to 3 months
- >3 months to 6 months
- >6 months

31. What intravenous antibiotic(s) would you give as an outpatient? (assumed to follow inpatient intravenous therapy unless 0 days of inpatient therapy are given)

- None
- Amoxycillin/Ampicillin
- Amoxycillin/clavulanate
- Benzylpenicillin
- Ceftaroline
- Ceftazidime
- Ceftriaxone
- Cefuroxime
- Cephazolin
- Clindamycin
- Flucloxacillin
- Ertapenem
- Gentamicin
- Lincomycin
- Linezolid
- Metronidazole
- Meropenem
- Piperacillin/tazobactam
- Ticarcillin/clavulanate
- Vancomycin

32. What would be the likely duration of outpatient intravenous antibiotics?

- 0 days
- 1 day to 3 days
- 4 days to 7 days
- 8 days to 14 days
- 15 days to 28 days
- >1 month to 2 months
- >2 months to 3 months
- >3 months to 6 months
- >6 months

33. How long after starting the first intravenous antibiotics would you be likely to start the oral antibiotics?

- The same day
- 1 day to 3 days
- 4 days to 7 days
- 8 days to 14 days
- 15 days to 28 days
- >1 month to 2 months
- >2 months to 3 months
- >3 months to 6 months
- >6 months

34. What oral antibiotics would you give? (Check all that apply)

- Amoxicillin
- Amoxycillin/ clavulanate
- Cephalexin
- Cefuroxime
- Ciprofloxacin
- Clindamycin
- Cotrimoxazole
- Di/Flucloxacillin
- Doxycycline
- Fusidic Acid
- Linezolid
- Metronidazole
- Moxifloxacin
- Phenoxymethylpenicillin
- Pristinamycin
- Rifampicin

35. What would be the likely duration of the oral antibiotics?

- 0 days
- 1 day to 3 days
- 4 days to 7 days
- 8 days to 14 days
- 15 days to 28 days
- >1 month to 2 months
- >2 months to 3 months
- >3 months to 6 months
- >6 months

***Scenario 1 - Oral antibiotics only***

You have chosen to give oral antibiotics only.

36. What oral antibiotics would you give? (Check all that apply)

- Amoxicillin
- Amoxycillin/ clavulanate
- Cephalexin
- Cefuroxime
- Ciprofloxacin
- Clindamycin
- Cotrimoxazole
- Di/Flucloxacillin
- Doxycycline
- Fusidic Acid
- Linezolid
- Metronidazole
- Moxifloxacin
- Phenoxymethylpenicillin
- Pristinamycin
- Rifampicin

37. What would be the likely duration of the oral antibiotics?

- 0 days
- 1 day to 3 days
- 4 days to 7 days
- 8 days to 14 days
- 15 days to 28 days
- >1 month to 2 months
- >2 months to 3 months
- >3 months to 6 months
- >6 months

**Scenario 1 - Different microbiology**

Imaging reveals no evidence of osteomyelitis but evidence of deep soft tissue infection. Surgical debridement is undertaken but residual infection remains with non-debrided deep soft tissue samples growing only methicillin sensitive S. aureus (MSSA) (penicillin-resistant). She is not known to be colonised by MRSA and there is a low prevalence of MRSA at your institution. Adherence is not thought likely to be an issue.

38. If only MSSA had been cultured from deep intra-operative soft tissue specimens would this change your treatment strategy?

- Yes
- No

**Scenario 2**

A highly functioning, independent 65 year old retired man with poorly controlled type 2 diabetes mellitus but no previous complications develops an ulcer overlying his 5th metatarsal head. After six weeks without treatment he attends your hospital and is found to have osteomyelitis of his 5^th^ metatarsal head. He is afebrile with an ESR of 75. There is evidence of peripheral neuropathy and moderate peripheral arterial disease with an ankle brachial index of 0.5. A CT angiogram reveals distal small vessel disease that cannot be corrected surgically or endovascularly. He has no allergies and has normal renal function. He is not known to be colonised with MRSA and there is a low prevalence of MRSA at your institution. He has previously been adherent to oral medication and is thought to be reliable with taking medication.

39. The patient is concerned amputation will impact on his golf and refuses amputation. He undergoes debridement of the ulcer and bone. Moderate growth of MSSA (penicillin resistant) is cultured from nondebrided deep tissue and direct microscopy reveals Gram positive cocci. The surgeon says that there is some residual infected bone and tissue but the bone appears healthy. What antibiotic strategy would you choose?

- Intravenous course only
- Initial intravenous course followed by oral therapy
- Concurrent intravenous and oral therapy or concurrent therapy followed by ongoing oral therapy
- Oral course only

***Scenario 2 - Intravenous antibiotics only***

You have chosen to give intravenous antibiotics only. Please identify below whether these would be given as an inpatient, outpatient or combination of these locations and what antibiotics you would use for what duration.

40. What intravenous antibiotic(s) would you give as an inpatient?

- None
- Amoxycillin/Ampicillin
- Amoxycillin/clavulanate
- Benzylpenicillin
- Ceftaroline
- Ceftazidime
- Ceftriaxone
- Cefuroxime
- Cephazolin
- Clindamycin
- Flucloxacillin
- Ertapenem
- Gentamicin
- Lincomycin
- Linezolid
- Metronidazole
- Meropenem
- Piperacillin/tazobactam
- Ticarcillin/clavulanate
- Vancomycin

41. What would be the likely duration of inpatient intravenous antibiotics?

- 0 days
- 1 day to 3 days
- 4 days to 7 days
- 8 days to 14 days
- 15 days to 28 days
- >1 month to 2 months
- >2 months to 3 months
- >3 months to 6 months
- >6 months

42. What intravenous antibiotic(s) would you give as an outpatient? (assumed to follow inpatient intravenous therapy unless 0 days of inpatient therapy are given)

- None
- Amoxycillin/Ampicillin
- Amoxycillin/clavulanate
- Benzylpenicillin
- Ceftaroline
- Ceftazidime
- Ceftriaxone
- Cefuroxime
- Cephazolin
- Clindamycin
- Flucloxacillin
- Ertapenem
- Gentamicin
- Lincomycin
- Linezolid
- Metronidazole
- Meropenem
- Piperacillin/tazobactam
- Ticarcillin/clavulanate
- Vancomycin

43. What would be the likely duration of outpatient intravenous antibiotics?

- 0 days
- 1 day to 3 days
- 4 days to 7 days
- 8 days to 14 days
- 15 days to 28 days
- >1 month to 2 months
- >2 months to 3 months
- >3 months to 6 months
- >6 months

***Scenario 2 - Intravenous antibiotics followed by oral antibiotics***

You have chosen to give intravenous antibiotics initially followed by oral antibiotics. Please identify below whether the intravenous antibiotics would be given as an inpatient, outpatient or combination of these locations and what antibiotics you would use for what duration.

44. What intravenous antibiotic(s) would you give as an inpatient?

- None
- Amoxycillin/Ampicillin
- Amoxycillin/clavulanate
- Benzylpenicillin
- Ceftaroline
- Ceftazidime
- Ceftriaxone
- Cefuroxime
- Cephazolin
- Clindamycin
- Flucloxacillin
- Ertapenem
- Gentamicin
- Lincomycin
- Linezolid
- Metronidazole
- Meropenem
- Piperacillin/tazobactam
- Ticarcillin/clavulanate
- Vancomycin

45. What would be the likely duration of inpatient intravenous antibiotics?

- 0 days
- 1 day to 3 days
- 4 days to 7 days
- 8 days to 14 days
- 15 days to 28 days
- >1 month to 2 months
- >2 months to 3 months
- >3 months to 6 months
- >6 months

46. What intravenous antibiotic(s) would you give as an outpatient? (assumed to follow inpatient intravenous therapy unless 0 days of inpatient therapy are given)

- None
- Amoxycillin/Ampicillin
- Amoxycillin/clavulanate
- Benzylpenicillin
- Ceftaroline
- Ceftazidime
- Ceftriaxone
- Cefuroxime
- Cephazolin
- Clindamycin
- Flucloxacillin
- Ertapenem
- Gentamicin
- Lincomycin
- Linezolid
- Metronidazole
- Meropenem
- Piperacillin/tazobactam
- Ticarcillin/clavulanate
- Vancomycin

47. What would be the likely duration of outpatient intravenous antibiotics?

- 0 days
- 1 day to 3 days
- 4 days to 7 days
- 8 days to 14 days
- 15 days to 28 days
- >1 month to 2 months
- >2 months to 3 months
- >3 months to 6 months
- >6 months

48. What oral antibiotics would you give after the intravenous antibiotics? (Check all that apply)

- Amoxicillin
- Amoxycillin/ clavulanate
- Cephalexin
- Cefuroxime
- Ciprofloxacin
- Clindamycin
- Cotrimoxazole
- Di/Flucloxacillin
- Doxycycline
- Fusidic Acid
- Linezolid
- Metronidazole
- Moxifloxacin
- Phenoxymethylpenicillin
- Pristinamycin
- Rifampicin

49. What would be the likely duration of the oral antibiotics?

- 0 days
- 1 day to 3 days
- 4 days to 7 days
- 8 days to 14 days
- 15 days to 28 days
- >1 month to 2 months
- >2 months to 3 months
- >3 months to 6 months
- >6 months

***Scenario 2 - Concurrent intravenous antibiotics and oral antibiotics***

You have chosen to give concurrent intravenous and oral antibiotics, or intravenous antibiotics followed by concurrent antibiotics. Please identify below whether the intravenous antibiotics would be given as an inpatient, outpatient or combination of these locations and what antibiotics you would use for what duration.

50. What intravenous antibiotic(s) would you give as an inpatient?

- None
- Amoxycillin/Ampicillin
- Amoxycillin/clavulanate
- Benzylpenicillin
- Ceftaroline
- Ceftazidime
- Ceftriaxone
- Cefuroxime
- Cephazolin
- Clindamycin
- Flucloxacillin
- Ertapenem
- Gentamicin
- Lincomycin
- Linezolid
- Metronidazole
- Meropenem
- Piperacillin/tazobactam
- Ticarcillin/clavulanate
- Vancomycin

51. What would be the likely duration of inpatient intravenous antibiotics?

- 0 days
- 1 day to 3 days
- 4 days to 7 days
- 8 days to 14 days
- 15 days to 28 days
- >1 month to 2 months
- >2 months to 3 months
- >3 months to 6 months
- >6 months

52. What intravenous antibiotic(s) would you give as an outpatient? (assumed to follow inpatient intravenous therapy unless 0 days of inpatient therapy are given)

- None
- Amoxycillin/Ampicillin
- Amoxycillin/clavulanate
- Benzylpenicillin
- Ceftaroline
- Ceftazidime
- Ceftriaxone
- Cefuroxime
- Cephazolin
- Clindamycin
- Flucloxacillin
- Ertapenem
- Gentamicin
- Lincomycin
- Linezolid
- Metronidazole
- Meropenem
- Piperacillin/tazobactam
- Ticarcillin/clavulanate
- Vancomycin

53. What would be the likely duration of outpatient intravenous antibiotics?

- 0 days
- 1 day to 3 days
- 4 days to 7 days
- 8 days to 14 days
- 15 days to 28 days
- >1 month to 2 months
- >2 months to 3 months
- >3 months to 6 months
- >6 months

54. How long after starting the first intravenous antibiotics would you be likely to start the oral antibiotics?

- The same day
- 1 day to 3 days
- 4 days to 7 days
- 8 days to 14 days
- 15 days to 28 days
- >1 month to 2 months
- >2 months to 3 months
- >3 months to 6 months
- >6 months

55. What oral antibiotics would you give? (Check all that apply)

- Amoxicillin
- Amoxycillin/ clavulanate
- Cephalexin
- Cefuroxime
- Ciprofloxacin
- Clindamycin
- Cotrimoxazole
- Di/Flucloxacillin
- Doxycycline
- Fusidic Acid
- Linezolid
- Metronidazole
- Moxifloxacin
- Phenoxymethylpenicillin
- Pristinamycin
- Rifampicin

56. What would be the likely duration of the oral antibiotics?

- 0 days
- 1 day to 3 days
- 4 days to 7 days
- 8 days to 14 days
- 15 days to 28 days
- >1 month to 2 months
- >2 months to 3 months
- >3 months to 6 months
- >6 months

***Scenario 2 - Oral antibiotics only***

You have chosen to give oral antibiotics only.

57. What oral antibiotics would you give? (Check all that apply)

- Amoxicillin
- Amoxycillin/ clavulanate
- Cephalexin
- Cefuroxime
- Ciprofloxacin
- Clindamycin
- Cotrimoxazole
- Di/Flucloxacillin
- Doxycycline
- Fusidic Acid
- Linezolid
- Metronidazole
- Moxifloxacin
- Phenoxymethylpenicillin
- Pristinamycin
- Rifampicin

58. What would be the likely duration of the oral antibiotics?

- 0 days
- 1 day to 3 days
- 4 days to 7 days
- 8 days to 14 days
- 15 days to 28 days
- >1 month to 2 months
- >2 months to 3 months
- >3 months to 6 months
- >6 months

**Treatment of diabetic foot infections**

59. For acute mild to moderate diabetic foot infection with no evidence of underlying bone or joint infection and no antimicrobial allergies, I prescribe EITHER (1) amoxicillin-clavulanate OR (2) cephalexin PLUS metronidazole:

- Never (0%)
- Very rarely (1 to 20%)
- Rarely (21 to 40%)
- Sometimes (41 to 60%)
- Often (61 to 80%)
- Very often (81 to 99%)
- Always (100%)

60. When treating patients with diabetic foot infections I ensure that I prescribe antibiotics that are active against organisms identified from superficial swabs:

- Never (0%)
- Very rarely (1 to 20%)
- Rarely (21 to 40%)
- Sometimes (41 to 60%)
- Often (61 to 80%)
- Very often (81 to 99%)
- Always (100%)

61. When treating patients with moderate to severe diabetic foot infections I empirically cover for MRSA:

- Never (0%)
- Very rarely (1 to 20%)
- Rarely (21 to 40%)
- Sometimes (41 to 60%)
- Often (61 to 80%)
- Very often (81 to 99%)
- Always (100%)

62. How would you describe the prevalence of MRSA amongst diabetic foot patients at your predominant institution?

- Low (<5%)
- Intermediate (5 to 20%)
- High (>20%)
- I don't know

63. The ASID Clinical Research Network has established the DEFIANZ group to improve the management of diabetic foot infections through multi-centre clinical research. What suggestions do you have for one or more binational studies in diabetic foot infections in Australia and New Zealand?
